# Industrial linkage effects of RCEP economies’ imports of producer services on manufacturing advantages

**DOI:** 10.1371/journal.pone.0253823

**Published:** 2021-07-15

**Authors:** Ying Qiu, Yushuang Gong

**Affiliations:** 1 School of Economics, Jiangxi University of Finance and Economics, Nanchang, China; 2 Research Institute for Global Value Chains, University of International Business and Economics, Beijing, China; 3 Research Institute of Industrial Economics at the Jinan University, Jinan, China; Szechenyi Istvan University: Szechenyi Istvan Egyetem, HUNGARY

## Abstract

This study examines the industrial transmission mechanisms between producer services imports and manufacturing advantages in RCEP economies. Based on the framework of GVCs’decomposition, we establish a hierarchical linear model (HLM) withthe data from 2007 to 2017 in the ADB MIRO, WTO BaTiS and WITS databases to analyze the impact of producer services imports in RCEP economies on the value-added domestic exports of manufacturing in countries around the world, and the conclusions are as follows: (1) Producer service imports have a significant positive impact on the domestic value-added of manufacturing exports through direct effects, upstream effects and downstream effects. The downstream effect is the main driving force for the improvement of manufacturing. After using instrumental variables to resolve endogeneity, the conclusion remains stable; (2) The downstream effect of insurance imports is the largest among producer services imports. After the outbreak of the world financial crisis, the transmission effect of the industry chain was higher in 2008 than before the crisis. (3) Due to the high degree of specialization of industrial division in developed economies, the transfer effect of the three major industrial chains is greater than that of developing countries and regions.

## I. Introduction

Since being officially signed, RCEP has formed the world’s largest free trade agreement. In 2019, the GDP of 15 member countries reached 25.6 trillion US dollars, accounting for 29.3% of the global economy. Total value of intra-regional trade was 10.4 trillion US dollars, accounting for 27.4% of global trade [1]. Producer services such as finance, communication and computer services are increasingly becoming new engines to break through the low-end locking of the GVCs and reshape the comparative advantage of manufacturing because of their specialization, knowledge spillover effect and economies of scale. The import of producer services has a typical "Bypass Effect", i.e. bringing foreign high-quality services and their supporting advanced technologies and mature management experience, enriching the types and quality of service products in RCEP economies, promoting the expansion of these economies’ service markets.

**Fig 1:**
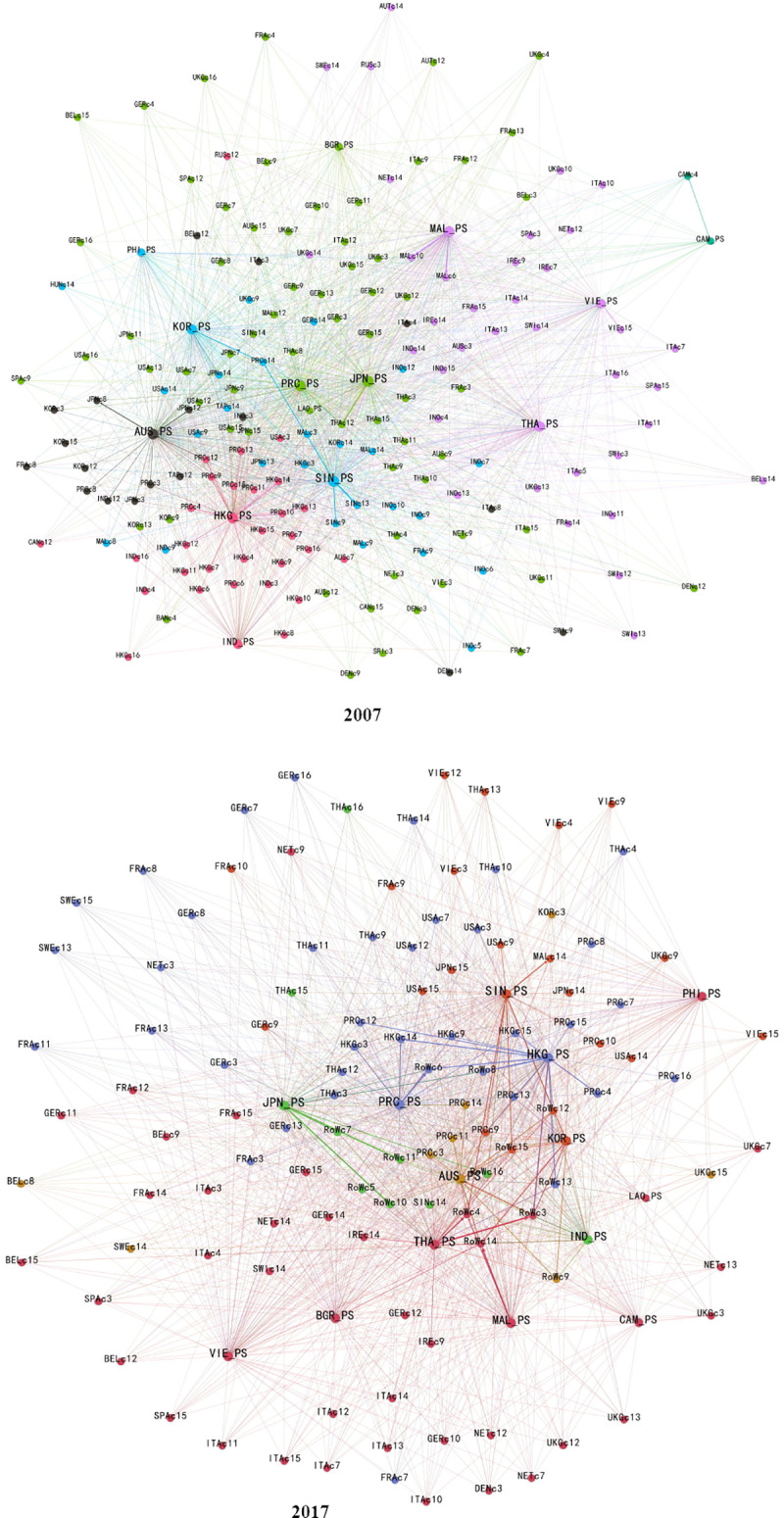
Comparison of the contribution of producer services from RCEP economies from 2007 to 2017. Source: ADB-MRIO database.

We used Gephi to draw a complex network analysis of the percentage of producer services in RCEP countries and regions participating in manufacturing in 62 countries and regions in the world, as shown in Fig 1. It can be seen from the figure that in 2007, China and Japan were dual centers in the RCEP regions. Producer services in Singapore, South Korea, and Australia also provided large support for manufacturing. By 2017, China’s producer services have become the core of the RCEP regions, and value added of imports within the region accounted for 55% in exports of the RCEP regions [2].

We divide the industrial transmission mechanism of import of producer services into three channels, i.e. direct effect, upstream effect and downstream effect, according to the decomposition framework of GVCs. Firstly, the direct effect means that the import of producer services has obvious positive effects on the local service market through knowledge spillover, product diversification and product quality improvement. The direct effect mainly comes from two aspects. The first is from economies of scale. Different from commodity trade, the biggest feature of service trade is that a large number of imported services need to be co-produced by local factors (labor and capital) in cooperation with foreign factors, i.e., limited trade. The import of producer services expands the local service market by introducing foreign enterprises to participate in competition, resulting in economies of scale, which brings significant direct effects [3], but the basic guarantee of this effect is that the importing country has a good business environment, transparent system and competition policy, so as to help foreign enterprises participate in its domestic competition equally [4]. The second is from technology spillover. If the foreign factors brought by the import of producer services are only a substitute for the local factors and do not lead to the expansion of market scale and the intensification of competition, there will be no obvious economies of scale, but the average production cost of local service enterprises can still be reduced through technology spillover or learning by doing, and the driving force for endogenous growth of local service industries can be enhanced. Relatively low barriers to service trade in RCEP economies are required for the generation of this effect, that is, encouraging the technology and knowledge transfer of foreign service enterprises by improving the liberalization of service trade.

Secondly, the upstream effect means that the import of producer services has a positive impact on the local initial factors and their related upstream industries of the value chain through backward industry association. First of all, producer services are customized. Therefore, in the choice of Mode 1 (cross-border delivery) and Cross-border 3 (commercial presence), the more high-end service providers tend to choose commercial presence to approach the final consumers, and the foreign elements will be jointly produced with local elements to meet the customized needs of high-end consumers. Thus, it will increase the demand for local factors of production and bring obvious "upstream spillover effect". Secondly, the producer services have the property of proximity constraint, so they only positively promote the industrial production network in the neighboring region, and their import reduces the cost of local service-related infrastructure. In particular, the import of related technologies such as digital trade has compositional effect on the value chain through the backward correlation effect. By transforming the traditional linear supply chain into an integrated supply chain ecosystem with data analysis as the core, and optimizing the accuracy of big data analysis and prediction through algorithms, the customer demand is tracked in real time, thus the governance structure of the upstream supply chain is reshaped from the perspective of backward relevance.

Thirdly, the downstream effect refers to the significant positive effect of service import on the development of downstream industries through the forward industry relevance effect. Downstream effects include: the first is the generated amplification effect. Producer services play two important roles in the value chain. One is at the high end of the value chain, that is, at both ends of the smiling curve, representing a higher source of value added [5]. The second is the link connecting the segmented production links in the value chain, connecting the discrete production processes into an integrated division of labor network [6]. Therefore, the service import cost reduction brought by trade liberalization will have an amplification effect through the industry association effect, especially infrastructure-related services (finance, communication, logistics, etc.), having a superposition effect on the cost reduction of the downstream manufacturing industry. The second is the generated compositional effect, that is, one of the important ways of importing producer services is offshore service outsourcing, which still belongs to the category of cross-border supply (Mode 1) because there is no shift between service providers and purchasers. Manufacturing enterprises importing producer services outsource their inefficient value chain links to foreign enterprises, focus on the core links of the enterprises and improve the overall productivity to generate the Compositional Effect. Especially, services such as outsourcing services of computer information technology are more helpful to make the production efficiency of the contract-issuing manufacturing enterprises approach the technological frontier [7].

The empirical analysis strategy of this paper is as follows: firstly, the decomposition framework of Wang et al. [8] is adopted based on the direct effect, upstream effect and downstream effect of the import of producer services, the value added trade measurement index is constructed from the direct competition channel, the upstream channel and the downstream channel respectively to investigate the impact of the import of producer services on the domestic value added in manufacturing export. Secondly, a multi-level linear model is established in this paper based on the WIOD (World Input-output Database) 2016, WTO BaTiS database and WITS (World Integrated Trade Solution) data, it has been proved that the import of producer services has a positive impact on the domestic value added in manufacturing exports through industrial association. Finally, in order to eliminate the potential endogenous problems of the model, we take the three effects of India’s imports of producer services as instrumental variables, and uses the instrumental variable method to conduct robustness verification.

We prove the following conclusions through empirical models: (1) the import of producer services has a significant positive impact on the domestic value added in RCEP economies’ manufacturing exports through three major effects, and the downstream effect of the import of producer services is the main force to improve the domestic value added in RCEP economies’ manufacturing exports; (2) the import effect of producer services has significant sectoral heterogeneity, the import of six producer services has different effects on RCEP economies’ domestic value added and the downstream effect of insurance services has the greatest positive impact; (3) after the financial crisis, the transmission effects of the three major industrial chains increased, indicating that the world financial crisis in 2008 does not have a negative impact on the industrial transmission effects of imports of RCEP economies’ producer services, but promotes the further expansion of the domestic value added in manufacturing exports; (4) compared with developing countries, RCEP economies’ imports of producer services from developed countries brings more significant industrial transmission effect.

The limitations of this paper are as follows: firstly, limitation of data. We can only find data from multinational input-output tables and international organization databases, which can not fully reflect the detailed mechanisms from multinational corporations. Secondly, lack of perspective of spatial analysis. There are geographical layout effect of producer services on exports of manufacturing besides the industrial transmission. We will pursue to explore how the spatial factors impact the causality between producer services imports and manufacturing exports.

## II. Literature review

This paper is related to the following literatures: Firstly, we extend the conclusions of relevant literatures with regard to the industry relevance effect of producer services. Producer services have four characteristics: intermediate input, customization, Internet intermediary and proximity constraint. Jones and Kierzkowski [6] were the first to pay attention to the relevance of producer services in production networks. Hoekman and Mattoo [9] further identified two functions of producer service in industry relevance: on the one hand, producer service plays a linking role for each production link in global value chain; on the other hand, it is also a high value added link and value added source on the smiling curve. Antràs and Gortari [10] constructed a multi-stage general equilibrium model of global value chain (GVC) and found that the best position of intermediate inputs of services and material objects in the global value chain is a function of a country’s marginal cost in this production stage and its geographical distance from neighboring production stages. Domestic scholars mainly analyze the industry relevance effect of service trade from the perspective of service trade opening-up. Sheng [11] made a quantitative evaluation and welfare analysis on the specific commitments of general service trade and its sub-sectors. Zhang et al. [12] used empirical methods to prove how service trade liberalization affects the production efficiency of manufacturing enterprises through outsourcing effect, compositional effect and technology promotion effect of service tasks. Wang and Zhang [13] supplemented the service trade negotiations in the order of opening-up. Lu and Xu [14] established a theoretical model based on transaction costs, and found the positive promoting effect of Internet on the development of downstream industries.

Secondly, we contribute to the relevant literatures on the value added trade account framework. Hummels et al. [15] introduced the concept and measurement method of vertical specialization, and created the value added trade account system. Koopman et al. [16] further developed the method used for measuring the trade with value added as methodology. Johnson and Noguera [17] calculated the ratio of value added exports to total exports by using GTAP database, and re-measured the multi-country bilateral trade balances including China-US trade deficit. KWW [18], WWZ [19] and Wang et al. [20] established a complete value added trade account system based on total exports, and integrated the trade decomposition equation for measuring value added into a unified account framework, especially separated the double counting part of total exports. Wang et al. [21] adopted two methods (forward decomposition of value added and backward decomposition of final products) and obtained four kinds of production activities: pure domestic production, traditional trade export, simple global value chain and complex global value chain. At the same time, the related literatures of the second type of measurement system focuses on building indexes based on the decomposition framework of value added trade, including global value chain participation, position index, upstream degree relative to the final consumer, or downstream degree index relative to the value added source and cross-border times (Fally; Antràs et al.; Miller and Temurshoev; Wang et al.,) [22–25]. Many domestic scholars mainly conducted empirical application based on the above value chain measurement framework, and further expanded from the global value chain to national value chain. Li and Pan [26] integrated the national value chain and the international value chain into a unified framework for the first time, and investigated the embedding mode of global value chain in China’s various regions from the perspective of value added. Ma and Li [27] used the world input-output table to calculate the proportion of domestic value added, the length of global value chain and the index of upstream degree produced by manufacturing sector.

Finally, we enrich the empirical results of related literature on the promotion of manufacturing industry by imports of producer services. Francois and Woerz [28] confirmed that service import has significant industry heterogeneity on manufacturing development, which will benefit technology-intensive industries and damage labor-intensive industries. But at the same time, it will also decrease the FDI in service industry, because there is substitution effect between import of service industry (Mode 1) and commercial presence (Mode 3). Wei et al. [29] confirmed from theoretical model and empirical results that a country can partially overcome the obstacles of underdeveloped domestic service industry by relying on more imports of producer services. In developing countries with high trade barriers in services, because foreign services and domestic services are not perfect substitutes, service imports have a bypass effect on domestic service market, which can directly promote RCA exports to downstream manufacturing industries by bypassing relatively inefficient domestic service industries, especially financial and commercial services. However, the bypass effect still depends on the development of domestic service industry, and the low efficiency of domestic service industry will reduce the promotion effect of foreign service imports. Francois and Manchin [30] found that the positive effect of service import on domestic export also depends on the quality of a country’s system and service-related infrastructure (logistics, communication, etc.).

The following contents of this paper are arranged as follows: The second part introduces the data sources and model building methods, and introduces in detail the measurement methods of three explanatory variables: direct effect, downstream effect and upstream effect. The third part is an empirical analysis of the substitution effect for imports of RCEP economies’ producer services. The fourth part summarizes the research issues, research methods and relevant conclusions, and puts forward corresponding policy suggestions.

## III. Measurement model and data description

### (I) Benchmark model

Firstly, we construct the measurement indexes of direct effect, upstream effect and downstream effect of trade in producer services in industrial transmission based on the decomposition framework of value added trade. Secondly, We use the hierarchical linear model (HLM) to construct the benchmark model to analyze the impact of producer services’ industrial linkage on domestic value added in export of RCEP economies’ manufacturing industry. Considering the differences between groups caused by the high-dimensional data structure of the WIOD, this paper adopts a multi-layer linear model to improve the estimation accuracy of the model, and divides the data into three levels for estimation by setting the national level ID and the sector level ID respectively. Finally, we establish the instrumental variables of the three major effect indexes respectively, and use two-stage least square estimation to alleviate the endogenous problem of the model.


$$\Delta DVA_{k,j,t}=\beta_{0}+\beta_{1}\Delta Direct_{k,j,t}+\beta_{2}\Delta Down_{k,j,t}+\beta_{3}\Delta Up_{k,j,t}+\Pi_{k,j,t}+\varepsilon_{k,j,t}$$ (1)

Among them, *k* indicates the *k*^th^ country (*k* = 1, …, N); *j* indicates the *j*^th^ sector (*j* = 1, …, M); *t* indicates the *t*^th^ year (*t* = 1, …, T);Δ*DVA*_*k*,*j*,*t*_ indicates the first difference of RCEP economies’ manufacturing industry in the *t*^th^ year under framework for decomposition of total trade relative to domestic value added in export in base period;Δ*Direct*_*k*,*j*,*t*_ measures the first difference of the *t*^th^ year relative to direct effect of import of producer services in base period; Δ*Up*_*k*,*j*,*t*_ measures the difference of the *t*^th^ year relative to direct effect of import of producer services in base period;Δ*Down*_*k*,*j*,*t*_ measures the difference of the *t*^th^ year relative to downstream effect of import of producer services in base period; three indexes jointly construct the index of industrial transmission mechanism of producer service import;∏_*k*,*j*,*t*_ is a series of control variables, including GDP per capita, capital stock per capita, and proportion of the tertiary industry in GDP of each country;*ε*_*k*,*j*,*t*_ is an error term.

### (II) Variable selection and index measurement

#### 1. Domestic value added (DVA) of manufacturing export under the framework for decomposition of total trade

According to the framework for decomposition of total trade flows proposed by Wang et al. [20], assuming that there are three countries: *S*, *R* and *T*, the domestic value added (DVA) part of bilateral intermediate product trade is decomposed based on the input-output table. *Z*^*sr*^ and *Y*^*sr*^ represent the parts of products from country *S* and used as intermediate inputs and final products by country *R* respectively, *VA*^s^ and *X*^s^ represent the value added and output of country *S*, and the total output *X*^r^ of country *R* is decomposed into outputs driven by different final products, then the following equation is obtained:

$$X^{r}=B^{rs}Y^{ss}+B^{rs}Y^{sr}+B^{rs}Y^{st}+B^{rr}Y^{rs}+B^{rr}Y^{rr}+B^{rr}Y^{rt}+B^{rt}Y^{ts}+B^{rt}Y^{tr}+B^{rt}Y^{tt}$$ (2)

If the value added coefficient *V*^*s*^≡*VA*^*s*^(*X*^*s*^)^-1^, then, define the complete value added coefficient:

$$V^{s}B^{ss}+V^{r}B^{rs}+V^{t}B^{ts}=u\;u=(1,1,\ldots ,\;1)$$ (3)

If *E*^r^ expresses the total export value of country *R*, *L*^*ss*^ = (*I*-*A*^*ss*^)^-1^ expresses the domestic Leontief inverse matrix of country *S*, and the intermediate export of country *S* to country *R* may be expressed as:

$$Z^{sr}=A^{sr}X^{r}=A^{sr}L^{rr}Y^{rr}+A^{sr}L^{rr}E^{r}$$ (4)

According to the above Eqs (2)–(4), the export *E*^*sr*^ from country *S* to country *R* can be divided into the following 16 items:

$$\begin{array}{l} E^{sr}=A^{sr}X^{r}+Y^{sr}\\ =(V^{s}B^{ss}{)}^{'}\#Y^{sr}+(V^{r}L^{ss}{)}^{'}\#(A^{sr}B^{rr}Y^{rr})+\\ (V^{t}L^{ss}{)}^{'}\#(A^{sr}B^{rt}Y^{tt})+(V^{s}B^{ss}{)}^{'}\#(A^{sr}B^{rr}Y^{rt})+\\ \left(V^{s}B^{ss}{)}^{'}\#(A^{sr}B^{rt}Y^{tr})+(V^{s}L^{ss}{)}^{'}\#(A^{sr}B^{rr}Y^{rs}\right)\\ +(V^{s}L^{ss}{)}^{'}\#(A^{sr}B^{rt}Y^{ts})+(V^{s}L^{ss}{)}^{'}\#(A^{sr}B^{rs}Y^{ss})\\ +(V^{s}L^{ss}{)}^{'}\#[A^{sr}B^{rs}(Y^{sr}+Y^{st})]\\ +(V^{s}B^{ss}-V^{s}L^{ss}{)}^{'}\#[A^{sr}B^{rs}(Y^{sr}+Y^{st})]\\ +(V^{s}B^{ss}-V^{s}L^{ss}{)}^{'}\#(A^{sr}X^{r})\\ +(V^{r}B^{rs}{)}^{'}\#Y^{st}+(V^{r}B^{rs}{)}^{'}\#(A^{sr}L^{rr}Y^{rr})\\ +(V^{r}B^{rs}{)}^{'}\#(A^{sr}L^{rr}E^{r})+(V^{t}B^{ts}{)}^{'}\#Y^{sr}\\ +(V^{t}B^{ts}{)}^{'}\#(A^{sr}L^{rr}Y^{rr})+(V^{t}B^{ts}{)}^{'}\#(A^{sr}L^{rr}E^{r}) \end{array}$$ (5)

Thereinto, the domestic value added (*DVA*) absorbed by foreign countries is the sum of the first five parts:

$$\begin{array}{l} DVA=(V^{s}B^{ss})'\#Y^{sr}+(V^{s}L^{ss})'\#(A^{sr}B^{rr}Y^{rr})+(V^{s}L^{ss})'\#(A^{sr}B^{rt}Y^{tt})\\ \;+(V^{s}L^{ss})'\#(A^{sr}B^{rr}Y^{rt})+(V^{s}L^{ss})'\#(A^{sr}B^{rt}Y^{tr}) \end{array}$$ (6)

Part 1 is the domestic value added in final export; Part 2 is the domestic value added in intermediate exports directly absorbed by importing countries in order to produce domestic final demand; Part 3 is the domestic value added in intermediate exports exported to a third country by the importing country and absorbed by the third country in order to produce final domestic demand; Part 4 is the domestic value added in intermediate exports produced by importing countries, finally exported to and absorbed by the third country; Part 5 is the domestic value added in intermediate exports produced by importing countries and exported to the third country, and returned to the second country in final import for absorption.

#### 2. Direct effect of imports of producer services

According to the calculation method of Wang et al. [8], the direct effect is measured by the import penetration rate (Acemoglu et al., [31]), and import penetration rate reflects the proportion of imports of a certain industry (product) in a country’s total consumption. The direct effect is defined as the annual change rate of the import of RCEP economies’ sector *j* from country *k* to the total input of the sector from 2000 to the *t*^th^ year.


$$\Delta Direct_{k,j,t}=\frac{100}{t-2000}\times \frac{M_{j,t}^{k,RCEP}-M_{j,2000}^{k,RCEP}}{Y_{j,2000}^{RCEP}+M_{j,2000}^{*RCEP}-E_{j,2000}^{RCEP*}}$$ (7)

$M_{j,2000}^{k,RCEP}$ indicates the total import of RCEP economies’ producer service sector *j* from country *k* in 2000; $M_{j,2000}^{RCEP*}$ and $E_{j,2000}^{RCEP*}$ indicates the total import and export of RCEP Economies’ sector *j* from other countries in 2000 respectively; $Y_{j,2000}^{RCEP}$ indicates the total output of RCEP economies’ producer service sector *j* in 2000; $M_{j,t}^{*RCEP}-E_{j,t}^{RCEP*}$ indicates the total net import of the sector *j*; the numerator indicates the change of import of RCEP economies’ sector *j* from country *k* in the tth year relative to 2000; the denominator indicating the total input of the sector *j* in 2000.

#### 3. Upstream effect of imports of producer services

Upstream effect indicates that as the upstream sector of the industrial chain, sector *j* of producer services is indirectly affected by importing producer services *g* from country *k*. This index measures the annual change rate (from 2000 to the *t*^th^ year) of weighted average import penetration rate of the producer service industry *g* purchasing intermediate products from sector *j*:

$$\Delta UP_{k,j,t}=w_{j,g,2000}^{UP}\Delta Direct_{k,g,t}$$ (8)

$$w_{j,g,2000}^{UP}=\frac{Z_{j,g,2000}^{RCEP,RCEP}}{\sum_{i}Z_{j,i,2000}^{C,C}}$$ (9)

$Z_{j,g,2000}^{RCEP,RCEP}$ indicates that RCEP economies’ sector *g* purchases the output of domestic sector *j* as intermediate input, the weight $W_{j,g,2000}^{UP}$ indicates the percentage of intermediate products purchased by Chin’s sector *g* from sector *j* in the total sales of sector *j* in 2000. The higher the weight $w_{j,g,2000}^{UP}$ is, the greater will be the upstream effect of the import impact of sector *j* on the sector *g*.

#### 4. Downstream effects of imports of producer services

Downstream effect refers to the indirect impact on the imports of producer service *g* from country *k*, which is located in sector *j* as the downstream industry chain, which is constructed in two steps. Firstly, the fraction $\frac{M\_{\mathrm{int}}_{g,t}^{k,RCEP}-M\_{\mathrm{int}}_{g,2000}^{k,RCEP}}{Y\_{\mathrm{int}}_{g,2000}^{RCEP}+M\_{\mathrm{int}}_{g,2000}^{*RCEP}-E\_{\mathrm{int}}_{g,2000}^{RCEP*}}$ represents the weighted average of the impact faced by all departments *j* that purchase intermediate input g from country *k*. The index calculation is averaged annually, i.e., the first difference of year t relative to year 2000 is calculated on the basis of year 2000 every year, which is convenient for cross-year comparison.


$$\Delta Down_{k,j,t}=\frac{100}{t-2000}\times \sum_{g}w_{g,j,2000}^{Down}\frac{M\_{\mathrm{int}}_{g,t}^{k,RCEP}-M\_{\mathrm{int}}_{g,2000}^{k,RCEP}}{Y\_{\mathrm{int}}_{g,2000}^{RCEP}+M\_{\mathrm{int}}_{g,2000}^{*RCEP}-E\_{\mathrm{int}}_{g,2000}^{RCEP*}}$$ (10)

Where $M\_{\mathrm{int}}_{g,t}^{k,RCEP}$ represents the total amount of intermediate inputs imported by sector *g* of RCEP economies from country *k* in the *t*^th^ year, the numerator represents the variable of intermediate inputs imported by sector *g* of RCEP economies from country *k* in the *t*^th^ year relative to 2000, and the denominator is the total amount of intermediate inputs absorbed by sector *g* of RCEP economies in 2000. Secondly, construct the weight of sectors, which indicate the ratio of the purchasing amount of producer services sector *g* from country *k* by sector *j* in the downstream of RCEP economies’ industrial chain to the total purchasing amount in 2000.


$$W_{g,j,2000}^{Down}=\frac{Z_{g,j,2000}^{k,RCEP}}{\sum_{i}Z_{i,j,2000}^{k,RCEP}}$$ (11)

The numerator $Z_{g,j,2000}^{k,RCEP}$ in the weight indicates the total amount of intermediate inputs imported by sector *j* of RCEP economies from sector *g* of country *k* producer service in the base period, while the denominator $\sum_{i}Z_{i,j,2000}^{k,RCEP}$ indicates the total amount of all intermediate inputs from country *k* used by sector *j* of RCEP economies in the base period.

## IV. Estimation results and empirical analysis

### (I) Descriptive statistics

Import and export data of intermediate products come from world ADB-MRIO database, import and export data of service trade comes from WTO BaTiS database, and import and export data of goods trade comes from WITS (World Integrated Trade Solution) database, which covers 6 producer service sectors and 14 manufacturing sectors in 14 countries and regions from 2007 to 2017. The final product sector in the second quadrant of WIOD 2016 world input-output table does not divide the total trade separately, so the WITS database is selected for the total trade data. Descriptive statistics are as follows in Table 1.

**Table 1:** Descriptive statistics.

variable	N	mean	sd	min	max
**ΔDVA**	2,156	344.1614	754.4584	0.0061	3,102.86
**ΔDirect**	2,156	0.032	0.0471	-0.0237	0.1578
**ΔDownstream**	2,156	0.0009	0.0027	0	0.0121
**ΔUpstream**	2,156	0.001	0.0023	-0.0011	0.0091

It can be seen from Table 1 that the mean and fluctuation of the direct effect (ΔDirect) are the maximum, indicating that the direct effect is directly affected by the fluctuation of trade volume in the transmission effect of industrial chain caused by the import of producer services, with a large change range. The upstream effect and the downstream effect meet the theoretical expectation after industrial transmission.

### (II) Regression results of benchmark model

We use the maximum likelihood estimation method to estimate the multi-layer linear model (1), and the benchmark regression results are shown in Table 2.

**Table 2:** Maximum likelihood regression results of benchmark model.

	(1)	(2)	(3)	(4)	(5)
ΔDirect	0.049*** (7.57)	0.033*** (6.08)	0.005 (0.79)		0.012** (2.19)
ΔDownstream		1.248*** (30.49)		0.860*** (18.63)	0.866*** (18.76)
ΔUpstream			1.297*** (28.03)	0.807*** (16.41)	0.780*** (15.37)
Ser_Ratio	-0.050** (-2.54)	0.036** (2.20)	0.055*** (3.22)	0.072*** (4.56)	0.073*** (4.63)
PK	0.187*** (9.96)	0.171*** (10.90)	0.198*** (12.32)	0.183*** (12.28)	0.183*** (12.27)
PGDP	-0.009 (-0.23)	-0.03 (-0.90)	-0.123*** (-3.59)	-0.069** (-2.34)	-0.094*** (-2.97)
_cons	0.009** (2.56)	-0.003 (-1.20)	0.003 (1.01)	-0.005* (-1.89)	-0.003 (-1.12)
country id					
_cons	-5.623*** (-26.74)	-5.822*** (-28.23)	-5.582*** (-27.30)	-5.680*** (-28.78)	-5.793*** (-28.04)
Industry id					
_cons	-5.879*** (-94.31)	-5.944*** (-98.80)	-6.020*** (-97.08)	-6.023*** (-99.19)	-6.020*** (-99.20)

Residual	0.003975	0.003296	0.003395	0.003135	0.003134
ll	8654.792	9039.553	8989.396	9149.204	9151.415
aic	-17293.59	-18061.11	-17960.79	-18280.41	-18282.83
bic	-17248.18	-18010.02	-17909.71	-18229.33	-18226.07
N	2156	2156	2156	2156	2156

Note: p<0.1

** p<0.05

*** p<0.01.

Table 2 reports the test results of the three effects of imports of producer services in the benchmark model on the domestic value added in RCEP economies’ manufacturing exports. Column (1) reports the direct effect of imports of producer services, and the change of direct effect will increase the domestic value added in exports by 0.049, which is consistent with that in the existing literature. Columns (2) to (4) conduct pairwise regression for the three major channels respectively, and the results are all significantly positive, and significant at the 1% level. Column (5) reports the three major effects of the import of producer services on the domestic value added in exports of manufacturing. Where the direct effect is relatively small (0.012), while the maximum is the downstream effect (0.866), followed by the upstream effect (0.780), which fully proves that the imports of producer services must participate in RCEP economies’ manufacturing production process as an intermediate to enhance the upgrade of manufacturing. Only considering the influence of trade cannot fully identify the influence of producer services on the construction of domestic modern industrial system. From the characteristics of producer service, its function in the industrial chain is mainly to provide high-end technology and knowledge for the downstream manufacturing industry to reduce the manufacturing cost, and at the same time to transform the production factor structure and production function to improve the production efficiency of the upstream industry. Therefore, the upstream effect and the downstream effect are the key reasons for RCEP economies to successfully implement the import substitution strategy and promote the increase of the domestic added value of manufacturing exports.

## V. Expanding experience analysis

### (I) Heterogeneity analysis of producer service sector

According to the service sector classification published by the United Nations Balance of Payments Statistics, we classify 14 producer service sectors into six major sectors: transportation services, communication services, computer and information services, insurance services and other commercial activities. The regression results of the influence of producer service sub-sectors on the domestic value added in export of manufacturing are shown in Table 3. From Table 3, the empirical regression results of three major effects of all six major producer service sectors are basically consistent with the benchmark regression results of the overall samples, but showing obvious differences in size. In terms of direct effect, financial services have the highest degree of promoting the domestic value added in exports of manufacturing (0.232), but communication services and insurance services have no significant impact. In terms of upstream effect, the impacts of the six departments are all positive, among which the computer and information services have the greatest promotion effect (1.236) because of their impact on production factors and production functions. In terms of downstream effect, there is a big gap between the downstream manufacturing industry and the insurance service, while the Financial service and communication service are relatively small (0.841 and 0.622).

**Table 3:** Heterogeneity analysis results of producer service sub-sectors.

	Transport service	Communication service	Computer and information services	Financial service	Insurance services	Other commercial activities
ΔDirect	0.005* (1.71)	0.02 (0.52)	0.003 (1.42)	0.232*** (4.57)	-0.004 (-0.74)	0.01 (1.22)
ΔDownstream	0.567*** (8.64)	6.812*** (15.91)	31.711*** (16.82)	42.058*** (24.65)	197.786*** (16.37)	3.338*** (19.12)
ΔUpstream	0.970*** (21.81)	0.622*** (16.29)	1.236*** (20.73)	0.841*** (20.75)	1.123*** (23.20)	0.961*** (16.88)
_cons	0 (0.02)	-0.006** (-2.33)	-0.007** (-2.49)	-0.007** (-2.50)	-0.006** (-2.06)	-0.002 (-0.60)
Individual effect	Control	Control	Control	Control	Control	Control
Annual effect	Control	Control	Control	Control	Control	Control
Residual	0.003366	0.0033	0.003127	0.003133	0.003299	0.00307
N	2156	2156	2156	2156	2156	2156

Note

* p < 0.1

** p < 0.05

*** p < 0.01.

### (II) Heterogeneity analysis before and after the financial crisis

The time span of data is from 2007 to 2017. Therefore, the global financial crisis in 2008 is selected as the division point to investigate the heterogeneity of industrial relevance effect of imports of producer services before and after the financial crisis. As shown in Table 4, after the financial crisis, the three major effects of import of producer services generally increased compared with that before the financial crisis by 18.2%, 143.61% and 68.42% respectively, and the downstream effect and upstream effect coefficient reach about 1. Among them, the downstream effect coefficient and the increasing range are the largest. It shows that after the global financial crisis, the domestic division system of RCEP economies’ manufacturing industry has become more mature, especially the length of downstream industries has increased, the relevance of related industries has improved, and the economic resilience has become stronger and stronger. Therefore, the technological spillovers of imports of producer services and its linking role have been significantly enhanced. It can be seen that the global financial crisis has become an opportunity to improve the domestic industrial system and enhance the transmission effect of the industrial chain.

**Table 4:** Heterogeneity analysis result before and after the financial crisis.

	Before financial crisis	After financial crisis
ΔDirect	0.011*** (5.67)	0.013*** (4.64)
ΔDownstream	0.415*** (6.58)	1.011*** (10.14)
ΔUpstream	0.551*** (9.48)	0.928*** (11.73)
_cons	-0.002 (-0.63)	-0.013*** (-3.11)
Individual effect	Control	Control
Annual effect	Control	Control
N	196	1960

Note

* p < 0.1

** p < 0.05

*** p < 0.01.

### (III) Heterogeneity analysis between developed countries and developing countries

The heterogeneity analysis results of developed countries and developing countries are shown in Table 5. There are significant differences in the industrial transmission effect of RCEP economies’ import of producer services from different types of countries: among them, the direct effect and downstream effect of the import of producer services from developing countries are both greater, because most of RCEP economies’ imports of producer services from developing countries are labour intensive services, which can supplement the domestic lack of low skilled services and reduce the production cost of manufacturing industry. Especially for coastal areas deeply embedded in the division of the global value chain, the downstream manufacturing is benefited greatly from the import of producer services. However, the import of producer services from developed countries significantly improves the upstream effect, while the upstream effect from developing countries is not obvious. This shows that RCEP economies’ major imports of technology intensive and knowledge intensive service intermediates from developed countries can significantly improve the research, development and design of the upstream of the value chain and enhance the efficiency of production factor, while the upstream effect of developing countries is not significant, which also reflects the differences in the industrial structure and trade structure of different exporting countries of producer services among RCEP economies’ trade partners.

**Table 5:** Heterogeneity analysis results of developed countries and developing countries.

	Developing countries	Developed countries
ΔDirect	0.003 (0.48)	0.028*** (6.03)
ΔDownstream	0.644*** (11.45)	1.192*** (15.19)
ΔUpstream	1.180*** (17.29)	-0.203*** (-4.90)
_cons	-0.009*** (-2.90)	-0.001 (-0.17)
Individual effect	Control	Control
Annual effect	Control	Control
N	1540	616

Note: * p < 0.1, ** p < 0.05

*** p < 0.01.

### (IV) Endogeneity processing

We use the two stage least square (2SLS) for endogeneity processing for the model, and the methods of Arnold et al. [32] and Sun et al. [33] are followed. We intend to use the direct effect index, upstream effect index and downstream effect index of India’s imports of producer services from various countries in the world as instrumental variables. India’s service industry was opened earlier than RCEP economies. In the middle of the 1990s, India opened its service industry under the pressure of the IMF, with an emphasis on canceling the entry restrictions on foreign competitors. India’s imports of producer services and domestic value added in RCEP economies’ manufacturing exports are independent of each other, but the two countries are in a certain competitive position in the global economy. India’s imports of producer services may affect RCEP economies’ imports of producer services. Therefore, it is reasonable to use the industrial transmission index of India’s imports of producer services to construct instrumental variables. See Table 6 for specific results. The endogenous processing results show that the conclusions of the benchmark model remain robust. Except that the direct effect of importing producer services from developing countries is not significant, the coefficients of the main variables remain unchanged or slightly improved compared with the multi-layer linear regression model, which shows that the results of the benchmark regression model are robust.

**Table 6:** Regression results of instrumental variable method.

	Total sample	Before 2008	After 2008	Developed countries	Developing countries
ΔDirect	0.005*** (2.87)	0.002*** (3.80)	0.011*** (3.80)	0.008*** (5.71)	0.014 (1.57)
ΔDownstream	0.949*** (6.11)	0.658*** (5.14)	1.227*** (5.14)	0.811*** (8.11)	2.269** (2.80)
ΔUpstream	1.939*** 0.005***	2.163*** (4.88)	2.163*** (4.88)	1.801*** (7.30)	1.163** (2.67)
_cons	-0.003 (-0.69)	0.010* (1.72)	0.010* (1.72)	0 (0.02)	-0.003 (-0.57)
*R*^2^	0.634	0.637	0.637	0.644	0.54
N	2156	196	1960	616	1540

Note

* p < 0.1

** p < 0.05

*** p < 0.01.

The first stage regression results of instrumental variable method are reported in Table 7. It can be seen from the results that India’s imports of producer services and RCEP economies’ imports of producer services conform to the hypothesis of correlation, F statistics pass the test, and instrumental variable method is effective.

**Table 7:** First stage results of instrumental variable method.

	ΔDirect	ΔDownstream	ΔUpstream
ΔDirect(IV)	0.524*** (38.54)	0.001*** (5.68)	0.007*** (14.43)
ΔDownstream(IV)	1.368*** (7.46)	0.177*** (25.43)	0.087*** (11.79)
ΔUpstream(IV)	0.11 (0.13)	0.079** (2.67)	0.490*** (10.85)
First Stage F Statistics	311.05	238.17	199.45

## VI. Conclusions and suggestions

### (I) Conclusions

Through literature review, theoretical study and empirical analysis, we make an in-depth study on the effects of imports of producer services, and discusses the impact of imports of producer services on the domestic value added in RCEP economies’ manufacturing exports through direct effects, upstream effects and downstream effects. Through the construction of multi-layer linear model and extended analysis, we draw the following conclusions on the basis of existing study:

#### 1. The industrial transmission effect of imports of producer services has significant positive effect

At the same time, the imports of producer services have a positive impact on the domestic value added in RCEP economies’ manufacturing exports through three major channels of direct effect, downstream effect and upstream effect. The regression results of benchmark show that it is not comprehensive to only consider the direct effect of producer services on manufacturing industry in the past. The upstream effect and downstream effect of imports of producer services are significantly greater than the direct effect, and the downstream effect shall be the maximum. This result shows that the reason for RCEP economies’ successful implementation of import substitution strategy is not only due to the trade itself, but also because the service imports are deeply embedded in RCEP economies’ industrial system, which has become an indispensable part of domestic upstream and downstream industries, thus promoting the "internal circulation" of RCEP economies’ manufacturing industry and playing a significant positive role in building RCEP economies’ domestic integrated market. This conclusion remains robust after endogeneity processing by instrumental variable method.

#### 2. The industrial transmission mechanism of the imports of producer services is of significant heterogeneity

Firstly, from the subdivision of producer services, financial services are affected the most in term of direct effect, so are insurance services and computer and information technology services at downstream and upstream respectively. Secondly, from the perspective of time, the global financial crisis in 2008 not only does not weaken the industrial transmission effect of the import of producer services, but promotes the domestic manufacturing industry to further strengthen industrial linkages and "cultivate domestic strength", and the three major effects are greatly improved compared with those before the crisis. Thirdly, from the perspective of exporting countries, RCEP economies’ imports of labor-intensive services from developing countries has significantly improved direct effect and downstream effect, which reduces the manufacturing cost, while the imports of technology-intensive services from developed countries has significantly improved upstream effect.

### (II) Policy suggestions

The imports of producer services have a significant positive impact on the domestic value added in exports of RCEP economies’ manufacturing industry. Therefore, in order to give full play to the positive role of the imports of producer services, we put forward the following policy suggestions:

#### 1. Continuing to increase efforts to encourage the imports of producer services

According to RCEP economies’ strategic goal of constructing a "manufacturing power", high-end producer services are the key link and important component of high-quality development of manufacturing industry. RCEP economies shall further increase the imports of high-tech producer services such as R&D design, industrial design, business consulting, inspection and certification, and make a strong supplement to domestic producer services, so as to promote RCEP economies’ manufacturing industry to continuously advance to the high end of the value chain.

#### 2. Encouraging the deep integration of imports of producer services and advanced manufacturing industries

The imports of producer services promote the "internal circulation" of RCEP economies’ manufacturing industry, not only by the scale of trade, but also by the degree of relevance between imports and domestic industrial systems. RCEP economies shall further promote the deep integration of imports of producer services and advanced manufacturing industries, deepen business relevance, pay special attention to introducing high-end imports of producer services into the construction of regional industrial chains to "supplement" and "strengthen" the chain, and give full play to the bypass effect of imports of producer services to improve RCEP economies’ modern industrial system.

#### 3. Attaching significant importance to the role of imports of producer services in constructing a high-quality factor market

Existing policies and theories often focus on the downstream effect of imports of producer services on the high-quality development of manufacturing industry, i.e. technology spillover effect. However, empirical evidence shows that the upstream and downstream effects of imports of producer services are equally significant, which fully demonstrates the great potential of producer services in the upstream of the industrial chain. RCEP economies shall closely combine the imports of producer services with the innovation-driven strategy, strive to integrate the imports of high-end services with technological R&D innovation, improve the efficiency of upstream factor allocation, promote the innovative development of finance, information data, human resources and other services for domestic supply chain, and construct a Chinese manufacturing cluster with strong meta-innovation strength.
